# A dynamic pharmacometric framework defining the relationship between ivermectin exposure and mosquito lethality

**DOI:** 10.1038/s41598-026-58918-9

**Published:** 2026-07-18

**Authors:** Richard M. Hoglund, Kevin C. Kobylinski, Podjanee Jittamala, Borimas Hanboonkunupakarn, Narenrit Wamaket, Patchara Sriwichai, Siriporn Phasomkusolsil, Nicholas J. White, Nicholas P. J. Day, Joel Tarning

**Affiliations:** 1 Mahidol Oxford Tropical Medicine Research Unit, Faculty of Tropical Medicine, Mahidol University, Bangkok, Thailand; 2 Centre for Tropical Medicine and Global Health, Nuffield Department of Clinical Medicine, University of Oxford, Oxford, UK; 3 Department of Entomology, Armed Forces Research Institute of Medical Sciences, Bangkok, Thailand; 4 Department of Tropical Hygiene, Faculty of Tropical Medicine, Mahidol University, Bangkok, Thailand; 5 Department of Clinical Tropical Medicine, Faculty of Tropical Medicine, Mahidol University, Bangkok, Thailand; 6 Mahidol Vivax Research Unit, Faculty of Tropical Medicine, Mahidol University, Bangkok, Thailand; 7 Department of Medical Entomology, Faculty of Tropical Medicine, Mahidol University, Bangkok, Thailand

**Keywords:** Diseases, Drug discovery

## Abstract

Ivermectin is used in the treatment of several neglected tropical diseases. Studies have shown that it effectively kills anopheline mosquitoes. However, the relationship between the pharmacokinetic properties and mosquito-lethal effects have not been described quantitatively. Pharmacokinetic properties and mosquito-lethal effects associated with a single oral administration of ivermectin were evaluated in two healthy volunteer trials in Thailand. All data were pooled and analysed using nonlinear mixed-effect modelling. Ivermectin and metabolites were described by a parent-metabolite model. When co-administered with dihydroartemisinin-piperaquine, a reduced elimination clearance (41%) and a slower absorption (32%) of ivermectin was identified, resulting in higher exposures. Different peripheral volume of distributions of ivermectin in men and women were also observed (75% higher in females). Individual pharmacokinetic profiles were incorporated in a sigmoidal E_max_ model, which were used to quantify the relationship between ivermectin exposure and mosquito-lethal effects. The integrated models described successfully the observed mortality of both *Anopheles dirus* and *An. minimus*. The final models were used to illustrate the potential impact on vector-control associated with ivermectin administration. In conclusion, ivermectin and its metabolites showed effective mosquito-lethal effects. The developed pharmacometric framework could be a useful tool in the evaluation of ivermectin as a potential vector-control agent in malaria elimination campaigns.

**Supplementary Information:**

The online version contains supplementary material available at https://doi.org/10.1038/s41598-026-58918-9.

## Introduction

The latest report from the World Health Organization estimated a total of 263 million malaria cases and 697,000 deaths in 2023^1^. Alarmingly, malaria control has stalled and transmission has increased in some areas in recent years. Physiological and behavioral resistance to the insecticides used on long-lasting insecticide-treated bednets and for indoor residual spraying has been documented in *Anopheles* mosquitoes, ^2–4^ and this warrants the development of novel vector control compounds and control measures. Malaria control has become more urgent with the development and spread of drug resistance to artemisinin-based combination therapies (ACTs), the current first-line treatment of uncomplicated *falciparum* malaria. Resistance to the artemisinin component was first reported in Southeast Asia and has recently been observed in Africa^5–7^. Resistance to both the artemisinin component and some of the partner drugs is now common in Southeast Asia and has resulted in unacceptably high failure rates with first-line therapies^8–12^.

The drug ivermectin has long been used to treat parasitic infections in both humans and animals, and has also been shown to effectively kill *Anopheles* mosquitoes when blood-fed on ivermectin-containing blood. Field evidence from Africa suggests that ivermectin mass drug administration (MDA) reduces transmission of *Plasmodium* parasites through its mosquito-lethal effects^13–15^. A recent cluster randomized trial in Kenya demonstrated a reduction in clinical malaria episodes following three rounds of single-dose ivermectin (400 µg/kg) MDA^16^. However, ivermectin does not have any direct impact on the blood-stage or liver-stage malaria parasite at clinically relevant concentrations^17–20^. In the Greater Mekong Subregion, the two major malaria vectors are *Anopheles dirus* s.s. and *An. minimus* s.s., both of which are susceptible to ivermectin^21–24^.

Three major ivermectin metabolites have been identified and characterized in humans; 3”-*O*-demethyl ivermectin (M1), 4-hydroxymethyl ivermectin (M3), and 3”-*O*-demethyl, 4-hydroxymethyl ivermectin (M6)^25^. Biotransformation of ivermectin into these metabolites is achieved mainly through cytochrome P450 (CYP)3A4 and to a small extent by CYP3A5^25^. All three metabolites have shown to have mosquito-lethal effects comparable to ivermectin, when evaluated in membrane-feeding assays with *An. dirus*, *An. minimus*^24^, and *Anopheles stephensi*^26^. These results indicate that ivermectin metabolites and their pharmacokinetics should be taken into consideration when investigating the mosquito-lethal pharmacodynamic effects of ivermectin administration in humans.

We have reported previously on a clinical trial investigating the pharmacokinetics and mosquito lethal properties of ivermectin on *An. dirus* and *An. minimus*, and demonstrated effective killing of both species following membrane-feeding with blood from healthy human volunteers given a single oral dose of ivermectin (400 µg/kg) alone or in combination with the antimalarial drugs, dihydroartemisnin-piperaquine and primaquine^23^. The same study also showed increased drug exposure to ivermectin after co-administration with dihydroartemisinin-piperaquine (Study 1). However, pharmacokinetic data were not characterized and evaluated with a pharmacokinetic modelling approach, and mosquito-mortality was not incorporated into a simultaneous dynamic pharmacometric framework. This legacy data was combined with novel data from another healthy volunteer trial evaluating the pharmacokinetics of a single oral dose of ivermectin (400 µg/kg) and its metabolites as well as their mosquito-lethal effects through direct-feeding and membrane-feeding assays (Study 2).

The aim of the current study was to describe and quantify the pharmacological properties and mosquito-lethal effects of ivermectin and its three primary metabolites in healthy Thai volunteers treated with oral ivermectin, by developing a dynamic population-based pharmacokinetic-pharmacodynamic model.

## Results

Data from the two clinical trials, Study 1 and Study 2, were pooled and analysed using nonlinear mixed-effects modelling as implemented in Pumas AI v.2.6.1 (Pumas AI, USA)^27^. A total of 26 healthy Thai adult volunteers were included in this analysis. Study 1 consisted of 16 volunteers (56% females) with a median age of 26 (range: 29–56) years, and a median body weight of 62.9 kg (range: 48.3–73.5). Recruitment and subject follow-up in Study 1 was from September 2015 to September 2016. Primary safety outcomes for Study 1 were published previously^23^. Study 2 consisted of 10 volunteers (50% females) with a median age of 33 (range: 22–42) years, and a median body weight of 59.3 kg (range: 47.1–74.8). Recruitment and subject follow-up in Study 2 was from January to June 2019. No subjects were withdrawn from either Study 1 or Study 2. No notable treatment related adverse events or severe adverse events occurred in Study 2. A total of 1,135 venous blood samples and 190 venous plasma samples were collected from the volunteers and analysed for ivermectin and metabolite (M1, M3, and M6) concentrations, using a validated LC-MS/MS assay^24,28^. However, not all samples had a sufficient volume to be assayed for all compounds (*n* = 15).

### Pharmacokinetic modelling of ivermectin

A total of 1,148 ivermectin measurements were used in the analysis. Ivermectin pharmacokinetics were best described by a two-compartment disposition model. This model was superior to a one-compartment disposition model (*p* < 0.01), and a three-compartment disposition model did not improve the model fit (*p* > 0.05). The absorption of ivermectin was described by a transit compartment model consisting of three transit compartments, which resulted in the lowest objective function value.

### Pharmacokinetic modelling of metabolites

The developed ivermectin model was fixed using the derived individual estimates and imputed directly into the metabolite model. The metabolite model was based on 941 concentration measurements of M1, 988 concentration measurements of M3, and 766 concentration measurements of M6 above the lower limit of quantification (LLOQ; 0.2 ng/ml). Two M1 concentrations measurements from study 2 were excluded as outliers.

For the parent-metabolite structure it was assumed that ivermectin was completely metabolized to M1 and M3 in equal proportions, as this parameter is structurally unidentifiable from the observed data. Furthermore, it was assumed that M1 and M3 were both completely metabolized to M6, which was eliminated via an unknown pathway. Due to early high concentrations of M6, a direct metabolism pathway from ivermectin to M6 was implemented and the fraction of ivermectin undergoing this metabolism pathway was estimated. Two-compartment distribution models significantly improved the model fit for M1 (*p* < 0.001) and M3 (*p* < 0.001), but not for M6 (*p* > 0.05). Three-compartment models did result in an additional improvement for M1 (*p* < 0.001), but not for M3 (*p* > 0.05). However, when modelling the M1 metabolite alone, a three-compartment distribution model was not significantly better than a two-compartment model (*p* > 0.05). Therefore, for parsimony, M1 was kept as a 2-compartment model in the ivermectin-metabolite model. Metabolite M3 was kept as a 2-compartment model and M6 as a 1-compartment model.

### Simultaneous pharmacokinetic modelling of ivermectin and metabolites

The parent and metabolite data were successfully fitted simultaneously using the developed parent-metabolite model. This model was used to investigate the impact of drug-drug interaction and biological sex using a stepwise covariate search as implemented in Pumas AI for the forward step. The forward evaluation of drug-drug interaction as a covariate identified that concomitant dihydroartemisinin-piperaquine changed the elimination clearance and mean transit time while primaquine had an impact on the mean transit time and relative bioavailability. In the backward search only two of these were retained; dihydroartemisinin-piperaquine was found to reduce the elimination clearance of ivermectin (41.0% reduction) and increase the mean transit time (32.3%). Biological sex was investigated on the pharmacokinetic parameters of ivermectin. It was found that females had a 74.6% higher peripheral volume of distribution of ivermectin compared to males. The final model identified a proportional difference between blood and plasma concentrations, separately estimated for ivermectin and metabolites.

**Fig. 1:**
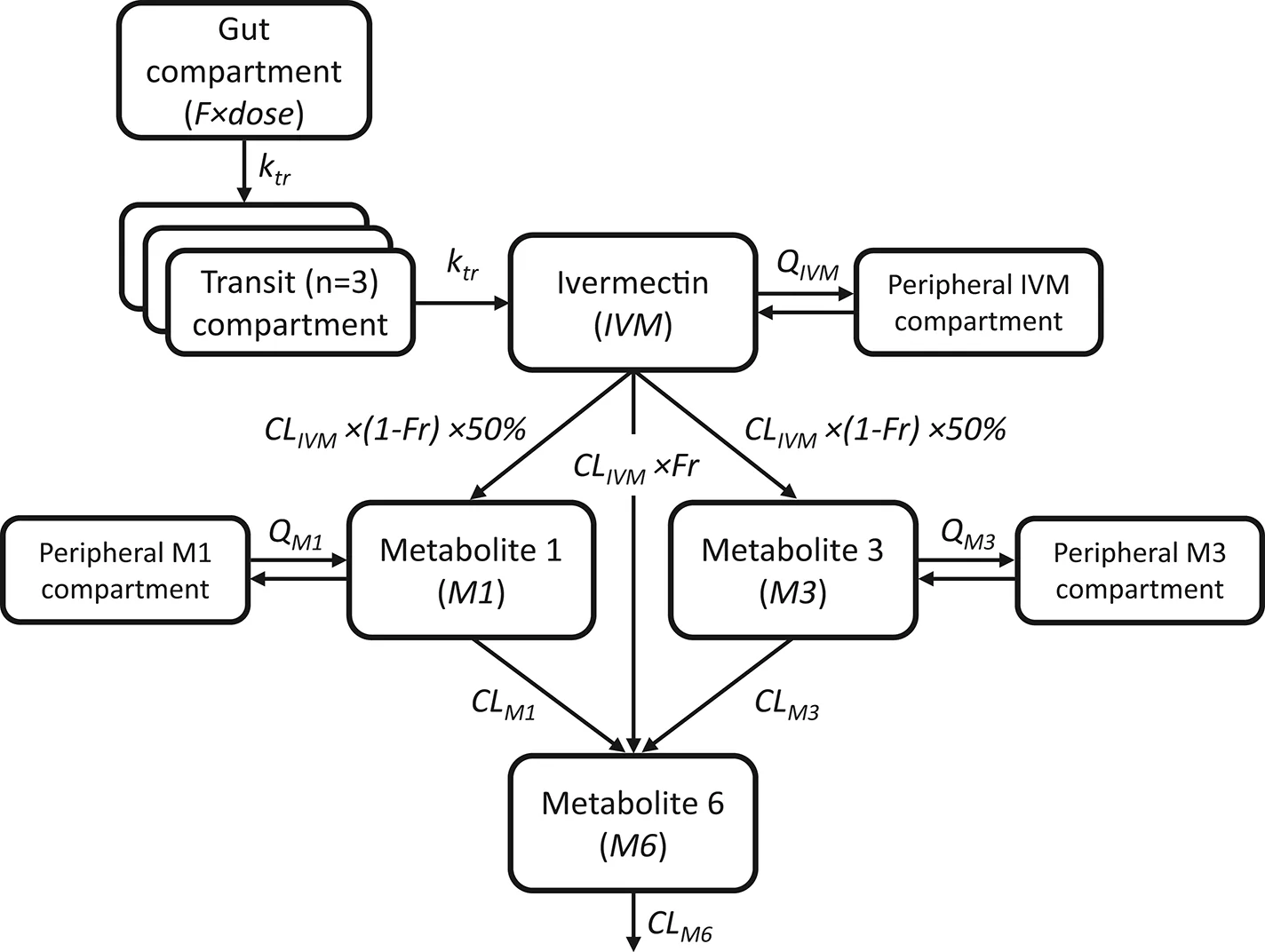
Schematic figure of the pharmacokinetic model for ivermectin (IVM) and its metabolites3”-*O*-demethyl ivermectin (M1), 4-hydroxyl ivermectin (M3), and 3”-*O*-demethyl, 4-hydroxyl ivermectin (M6). F is relative bioavailability. k_tr_ is the rate constant for the transit compartments. CL_IVM_ is the apparent elimination clearance of ivermectin. Q_IVM_ is the apparent inter-compartmental clearance for ivermectin. CL_M1_ is the apparent elimination clearance of M1. Q_M1_ is the apparent inter-compartmental clearance for M1. CL_M3_ is the apparent elimination clearance of M3. Q_M3_ is the apparent inter-compartmental clearance for M3. CL_M6_ is the apparent elimination clearance of M6.

**Fig. 2:**
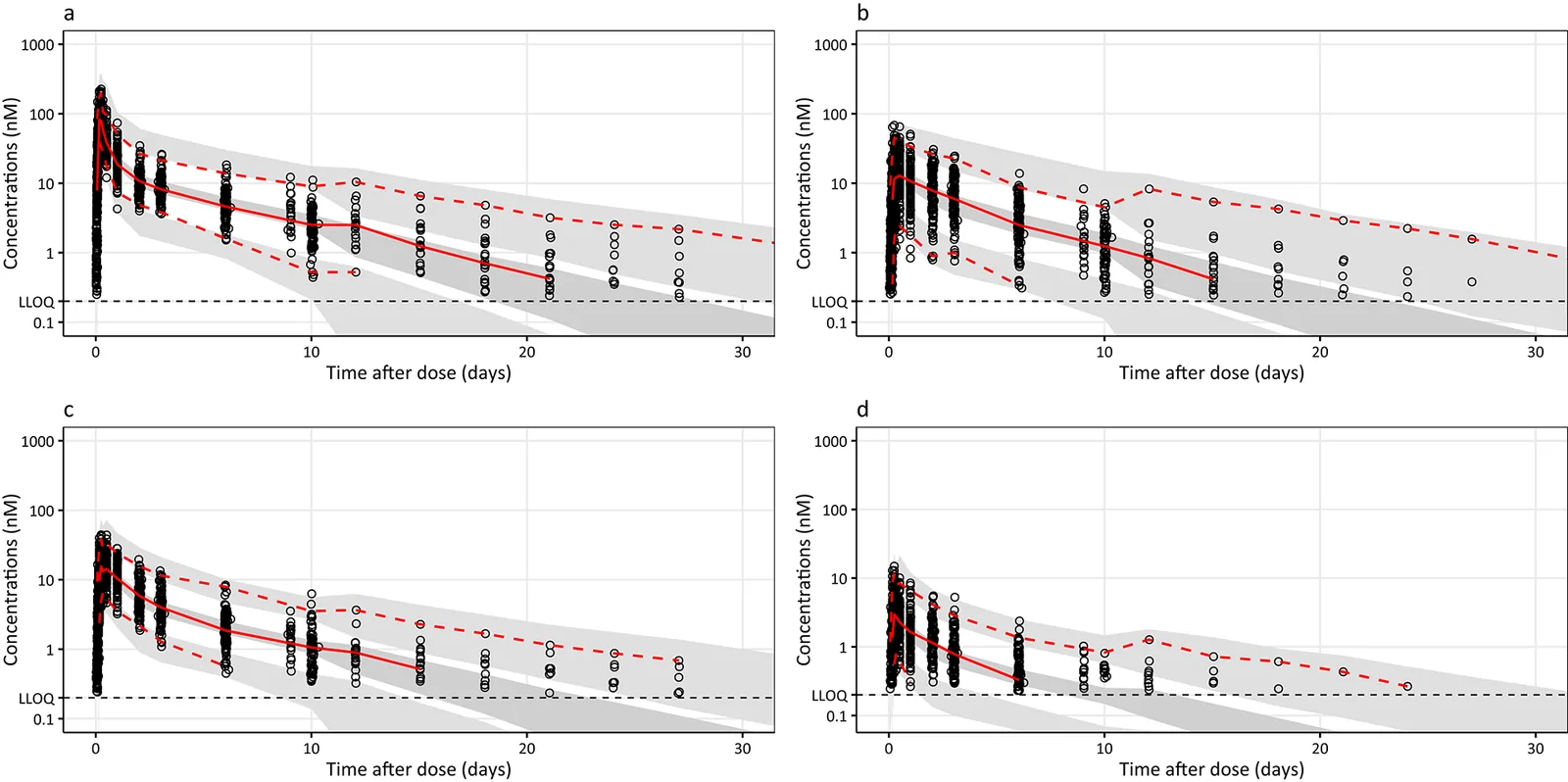
Visual predictive checks for ivermectin (plot a) and its metabolites3”-*O*-demethyl ivermectin (M1, plot b), 4-hydroxyl ivermectin (M3, plot c), and 3”-*O*-demethyl, 4-hydroxyl ivermectin (M6, plot d). Circles are observed concentrations. The solid red line is the median of the observed concentrations (50th percentile). The upper dashed red line is the 97.5th percentile of the observed data and the lower dashed red line is the 2.5th percentile of the observed data. The gray shaded areas are the 95% confidence intervals of the simulated data at each percentile.

The structure of the complete model is presented in Fig. 1. Parameter estimates and covariate effects are presented in Table 1. Goodness-of-fit diagnostics showed good performance of the model and are presented in Figures S1-S4 (supplementary material). The simulation-based diagnostics showed good predictive performance of the model (Fig. 2).

**Table 1:** Final pharmacokinetic and pharmacodynamic parameters of the joint pharmacometric model.

	Parameter estimate [RSE]	95% CI	BSV (RSE)	95% CI	Shrinkage (%)
Pharmacokinetics Ivermectin					
F (%)	100 *fix*	-	0.0713 [28.6]	0.0430 to 0.121	3.00
MTT (h)	3.66 [4.87]	3.31 to 4.02	0.0559 [28.0]	0.0322 to 0.0925	1.11
N	3 *fix*	-	-	-	-
CL/F (L/h)	12.3 [6.58]	11.0 to 14.1	0.0351 [30.8]	0.0198 to 0.0640	5.38
V_c_/F(L)	211 [8.38]	179 to 250	-	-	-
Q/F (L/h)	17.6 [8.30]	15.2 to 20.9	-	-	-
V_p_/F (L)	616 [11.7]	501 to 770	0.119 [28.4]	0.0722 to 0.199	2.68
Fr (%)	23.9 [15.5]	16.4 to 30.8	-	-	-
DHA-PPQ_CL/F (%)	-41.0 [3.77]	-43.7 to -37.8	-	-	-
DHA-PPQ_MTT (%)	32.3 [7.26]	27.7 to 36.8	-	-	-
Sex_V_p_/F (%)	74.6 [33.6]	28.9 to 126	-	-	-
Scale_plasma_ (%)	86.4 [11.4]	67.9 to 107	-	-	-
σ	0.511 [2.15]	0.491 to 0.533	-	-	2.05
***Pharmacokinetics 3”-O-demethyl ivermectin (M1)***					
CL/F (L/h)	7.94 [11.6]	6.58 to 10.1	0.258 [27.6]	0.154 to 0.433	1.17
V_c_/F(L)	29.1 [22.3]	18.2 to 42.5	0.797 [31.2]	0.450 to 1.40	10.0
Q/F (L/h)	19.2 [17.7]	13.4 to 26.8	-	-	-
V_p_/F (L)	111 [11.6]	87.1 to 138	-	-	-
Scale_plasma_ (%)	-31.1 [14.4]	-38.8 to -21.3	-	-	-
σ	0.546 [2.24]	0.523 to 0.572	-	-	2.80
***Pharmacokinetics 4-hydroxyl ivermectin (M3)***					
CL/F (L/h)	10.3 [8.96]	8.79 to 12.3	0.0784 [31.0]	0.0442 to 0.139	5.51
V_c_/F(L)	21.6 [12.6]	17.0 to 27.4	0.126 [47.5]	0.0467 to 0.270	27.2
Q/F (L/h)	12.3 [15.7]	9.32 to 17.2	-	-	-
V_p_/F (L)	94.0 [12.2]	74.8 to 120	-	-	-
Scale_plasma_ (%)	75.7 [12.0]	59.0 to 93.5	-	-	-
σ	0.416 [2.34]	0.399 to 0.435	-	-	2.87
***Pharmacokinetics 3”-O-demethyl***,*** 4-hydroxyl ivermectin (M6)***					
CL/F (L/h)	158 [9.77]	132 to 191	0.158 [27.8]	0.102 to 0.266	2.87
V_c_/F(L)	93.8 [22.6]	53.4 to 139	-	-	-
Scale_plasma_ (%)	157 [10.1]	127 to 188	-	-	-
σ	0.448 [2.52]	0.427 to 0.471	-	-	2.27
***Pharmacodynamics An. dirus***					
E_max_ (%)	100 *fix*	-	-	-	-
EC_50_ (nM)	7.89 [2.89]	7.45 to 8.30	-	-	-
γ	6.05 [1.66]	5.85 to 6.24	-	-	-
σ_low_conc_	0.184 [3.71]	0.172 to 0.198	-	-	2.24
σ_high_conc_	0.000001 *fix*	-	-	-	-
κ	1 *fix*	-	-	-	-
C_cutoff_ (nM)	58.6 [0.432]	58.2 to 59.2	-	-	-
***Pharmacodynamics An. minimus***					
E_max_ (%)	100 *fix*	-	-	-	-
EC_50_ (nM)	1.29 [7.80]	1.12 to 1.51	-	-	-
γ	5.50 [2.95]	5.16 to 5.81	-	-	-
σ_low_conc_	0.144 [12.6]	0.117 to 0.186	-	-	22.9
σ_high_conc_	0.000001 *fix*	-	-	-	-
κ	1 *fix*	-	-	-	-
C_cutoff_ (nM)	7.56 [2.64]	7.21 to 7.93	-	-	-

Parameters are presented for a 60 kg male volunteer with ivermectin given alone. CL/F is the apparent elimination clearance, V_c_/F is the apparent volume of distribution. Q/F is the apparent intercompartmental clearance. V_p_/F is the apparent peripheral volume of distribution. MTT is the mean transit absorption time. F is the relative bioavailability. Fr is the fraction of ivermectin being metabolized directly to metabolite M6. DHA-PPQ_CL/F is the change in ivermectin elimination clearance when dihydroartemisinin-piperaquine is given at the same time. DHA-PPQ_MTT is the change in ivermectin mean transit time when dihydroartemisinin-piperaquine is given at the same time. Sex_V_p_/F is the change in ivermectin apparent peripheral volume of distribution in females compared to males. Scale_plasma_ is the percentage change in individual predicted concentration in plasma compared to venous y blood. σ is the additive residual error given as standard deviation (equivalent to an exponential error as logarithmic pharmacokinetic data was modeled). σ_low_conc_ is an additive residual error used at concentrations lower than the cutoff. σ_high_conc_ is the additive residual error used at concentration above the cutoff. Κ is the slope factor in the transition between low and high residual error. C_cutoff_ is the cutoff concentration (sum of molar concentrations of parent and metabolites) for the transition between low and high residual error. The parameter estimates for *An. dirus* and *An. minimus* presented here are from whole blood ivermectin and metabolites. E_max_ is the maximum total effect. EC_50_, is the concentration resulting in 50% of maximum effect. γ is the shape factor in the sigmoidal E_max_-model. RSE is the percent relative error. 95% CI is the 95% confidence interval. BSV is the between-subject variability reported as variance. RSE and the corresponding 95% confidence interval was calculated with the sampling important resampling function, using 1,500 samples and 500 resamples.

### Pharmacokinetic-pharmacodynamic modelling

The pharmacodynamic measurement was defined as the proportion of dead mosquitoes at day 10 after they had fed on either volunteer blood through membrane feeding or directly on the volunteer. All pharmacodynamic data from study 1 and study 2 were pooled, and analyzed separately for *An. dirus* and *An. minimus*, using the individual estimates from the final pharmacokinetic parent-metabolite model. The pharmacodynamic mosquito-killing effect was described by a sigmoidal E_max_ model in which the sum of the molar venous whole-blood concentrations of ivermectin and its metabolites drives the effect. E_max_ was set to 100% as observed data demonstrated complete killing of mosquitoes at high concentrations, while EC_50_ and a shape factor were estimated in the applied models. Mosquito mortality at baseline was fixed to the observed individual values. To fully understand the difference in baseline mortality between the different studies, Kaplan-Meier analysis of the observed mosquito baseline mortality data was performed, comparing the membrane feeding assays of the two studies for *An. dirus* (Thailand strain) and *An. minimus*. The two studies showed significantly different survival at baseline for both *An. dirus* (Chi-Square = 23.3, p = < 0.001) and *An. minimus* (Chi-Square = 3.89, *p* = 0.0485) and is illustrated in Figure S5. Between subject variability was not included in any pharmacodynamic parameters as each individual blood sample (i.e. drug concentration) was associated with a mosquito killing activity derived from an independent ex-vivo experiment. Between subject variability was accounted for and maintained in the pharmacokinetic model. The final model included a concentration-varying residual with a smooth transition model using a logistic function, to account for the larger relative residual variability seen with lower mosquito mortality (i.e., count data). Covariates were investigated in the pharmacokinetic-pharmacodynamic models (e.g., mosquito feeding assay and strain of *An. dirus* mosquito). The code for the final pharmacokinetic-pharmacodynamic model is presented in the supplementary material.

### Anopheles dirus

**Fig. 3:**
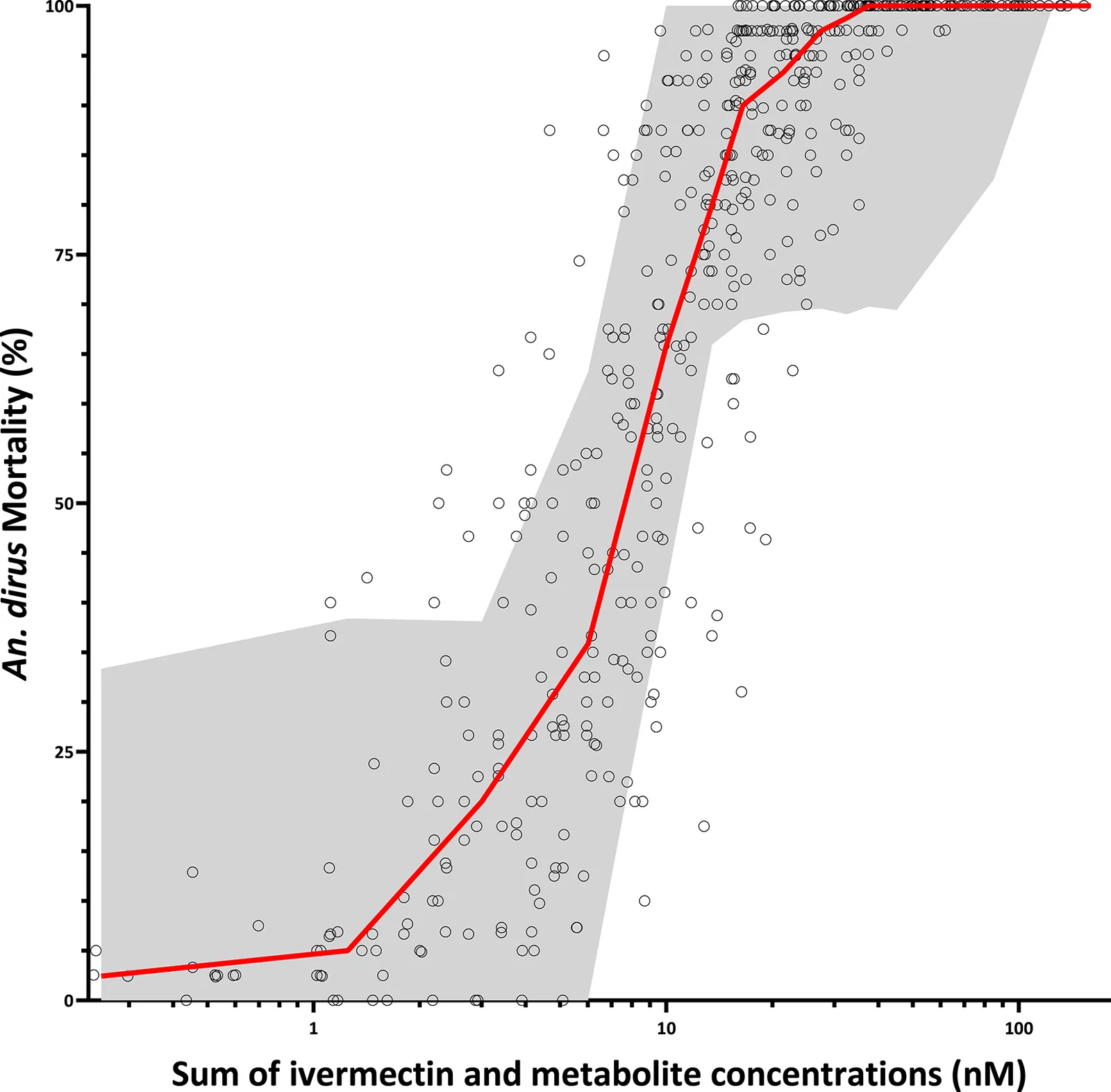
Visual predictive checks of *An. dirus* mosquito mortality. Circles are observed mortality associated with a individual blood sample. The solid red line is the median of the observed mortality data. The shaded area is the 95% confidence interval of the simulated data. The x-axis is truncated at the lower limit of quantification for ivermectin (0.230 nM) excluding 82 data points (including 46 baseline values).

A total of 618 *An. dirus* mortality measurements were used to develop the pharmacodynamic model. The killing of *An. dirus* mosquitoes was well described by the E_max_ model (whole blood EC_50_ of 7.89 (7.45–8.30)nM and a Hill factor of 6.05 (5.85–6.24)). The covariate search found no significant covariates (using a p-value of 0.01 in the forward search and 0.001 in the backward step). There were no substantial differences between the potency in killing *An. dirus* Thai strain (EC_50_ = 7.75 (7.26–8.24) nM) and Cambodian strain (EC_50_ = 8.89 (7.67–10.5) nM) which showed overlapping 95% confidence intervals, thus mortality data from both An. dirus strains were combined in the final analyses. The covariate search also found no significant difference for *An. dirus* EC_50_s between the different membrane and direct feeding assay methods. Further characterization of these outcomes will be provided in a separate manuscript. The parameter estimates for the final *An. dirus* whole blood ivermectin and metabolites model are presented in Table 1. The visual predictive check (Fig. 3) and goodness-of-fit plots (Figure S6) showed a good predictive performance and descriptive diagnostic fit of the final model. The estimated EC_50_ value is only associated with ivermectin and all three metabolites when measured in whole blood. Thus, for translational purposes, the final model was refitted with only ivermectin blood concentrations driving the effect, ivermectin plasma concentrations driving the effect, as well as ivermectin and all metabolites plasma concentrations driving the effect. The results for these three models are presented in Table S1.

### Anopheles minimus

**Fig. 4:**
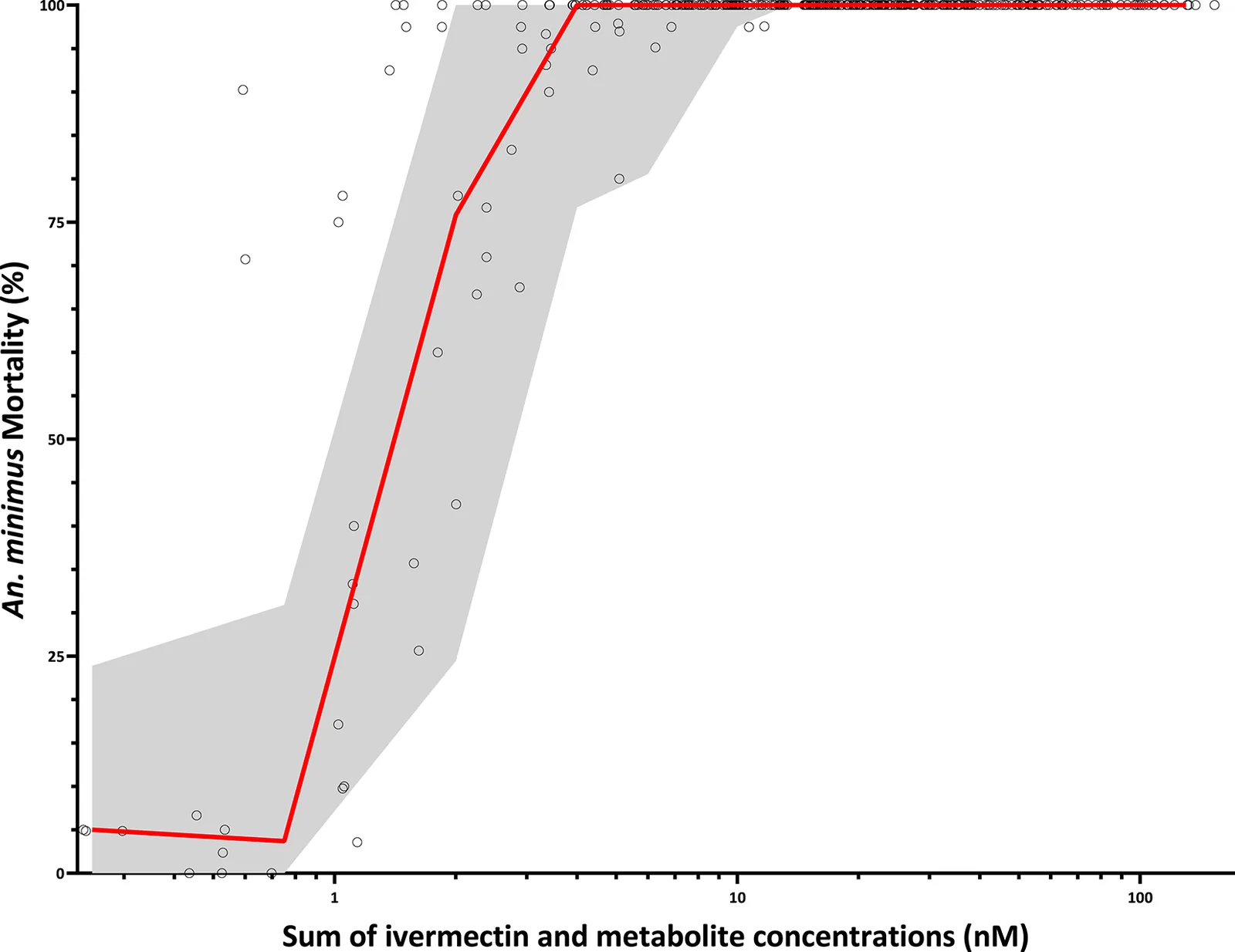
Visual predictive checks of *An. minimus* mosquito mortality. Circles are observed mortality associated with a individual blood sample. The solid black line is the median of the observed mortality data. The shaded area is the 95% confidence interval of the simulated data. The x-axis is truncated at the lower limit of quantification for ivermectin (0.230 nM) excluding 63 data points (including 26 baseline values).

A total of 523 *An. minimus* mortality measurements were used to develop the pharmacodynamic model. In study 2, half (five) of the volunteers were excluded from the analysis due to unexplainable high baseline mortality of mosquitoes fed on pre-treatment blood samples from membrane (40% baseline mortality) and direct (46% baseline mortality) mosquito feeds. However, enough data were available to develop a reliable pharmacodynamic model on the remaining data. The developed model described the killing of *An. minimus* mosquitoes, resulting in a substantially higher potency compared to *An. dirus* (whole blood EC_50_ of 1.29 (1.12–1.51) nM and a Hill factor of 5.50 (5.16–5.81)). The covariate search (using a p-value of 0.01 in the forward search and 0.001 in the backward step) found no significant difference for *An. minimus* EC_50_s between the membrane and direct feeding assay methods. Further characterization of these outcomes will be provided in a separate manuscript. The parameter estimates for the final *An. minimus* whole blood ivermectin and metabolites model are presented in Table 1. The visual predictive check (Fig. 4) and goodness-of-fit plots (Figure S7) showed a good predictive performance and descriptive diagnostic fit of the final model. As the estimated EC_50_ value is only relevant when ivermectin and all three metabolites have been measured in whole blood, the final model was refitted with only ivermectin blood concentrations driving the effect, ivermectin plasma concentrations driving the effect, as well as ivermectin and all metabolites concentrations driving the effect driving the effect. The results for these three models are presented in Table S1.

### Simulation of different field use cases

The simulated median concentration-time curves for ivermectin and its metabolites in a 60 kg male subject, after receiving a standard oral ivermectin dose, is shown in Figure S8 to illustrate the pharmacokinetic properties of these different compounds. The difference in ivermectin concentration-time curves and mosquito mortality between males and females is illustrated in Figure S9.

**Fig. 5:**
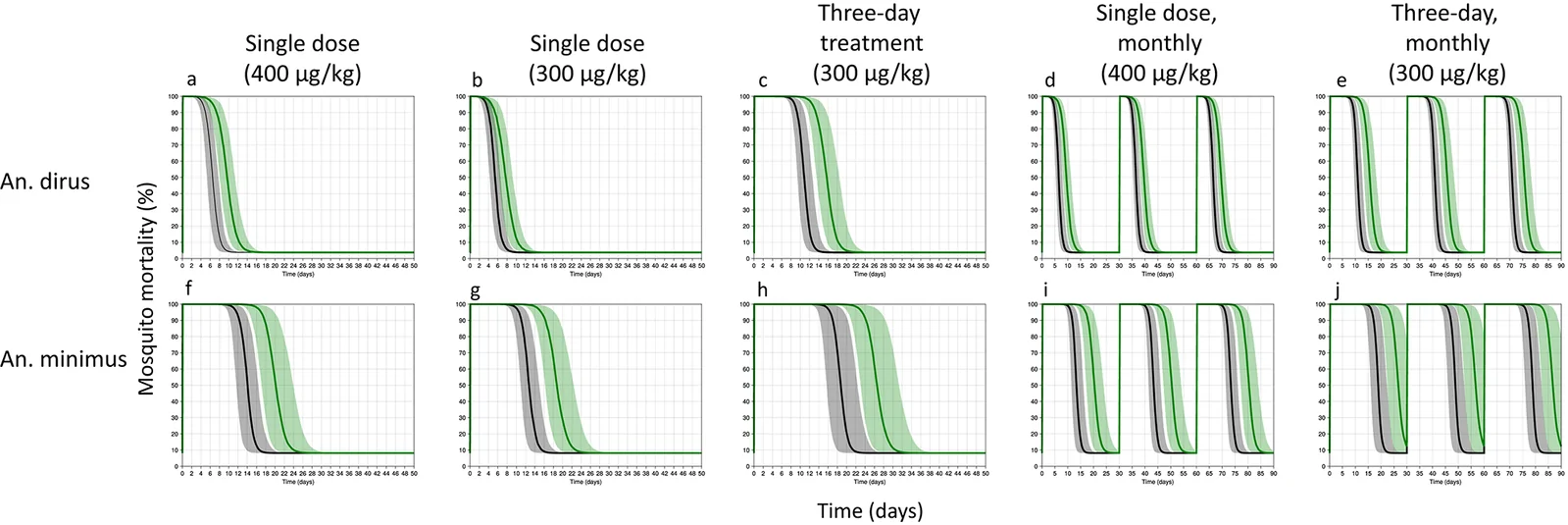
Simulated mosquito mortality against time for *An. dirus* (a-e) and *An. minimus* (f-j). The black curve is ivermectin given alone and the green curve is ivermectin given together with dihydroartemisinin-piperaquine. The shaded areas represent the 25th to 75th percentiles. Simulations were carried out for a 60 kg male volunteer.

To describe the mosquito mortality after different ivermectin administration strategies, the final pharmacokinetic-pharmacodynamic model was used to simulate (*n* = 500) a typical male subject of 60 kg, after different regimens of ivermectin given either alone or together with dihydroartemisinin-piperaquine. The investigated regimens were a single dose of ivermectin (either 300 µg/kg or 400 µg/kg), a three-day treatment once daily (300 µg/kg), a single dose of ivermectin (400 µg/kg) once a month for three months or a three-day treatment (300 µg/kg) once a month for three months. The standard single dose of ivermectin (400 µg/kg) showed a median sustained mosquito killing for 6.5 (*An. dirus*) and 14 (*An. minimus*) days when given alone and 9.6 (*An. dirus*) and 20 (*An. minimus*) days when given together with dihydroartemisinin-piperaquine. The simulated mosquito-mortality-time curves are presented in Fig. 5. Table 2 summarize the number of days the mortality is above 25%, 50%, and 90%. There was no substantial difference between 300 µg/kg and 400 µg/kg single oral dose with respect to mosquito mortality. However, the repeated ivermectin dosing and combinations with dihydroartemisinin-piperaquine substantially enhanced the mosquito killing effect.

**Table 2:** Simulated number of days above different mortality cut-offs.

	An. dirus						An. minimus					
	Alone			With DHA-PPQ			Alone			With DHA-PPQ		
	Time > 25%	Time > 50%	Time > 90%	Time > 25%	Time > 50%	Time > 90%	Time > 25%	Time > 50%	Time > 90%	Time > 25%	Time > 50%	Time > 90%
**Single dose** **(400 µg/kg)**	7.3 (6.0-8.6)	6.5 (5.2–7.7)	4.9 (3.9-6.0)	11 (9.0–13)	9.6 (8.0–11)	7.3 (5.9–8.9)	15 (12–17)	14 (12–16)	12 (10–14)	22 (18–26)	20 (17–24)	18 (15–21)
**Single dose** **(300 µg/kg)**	6.1 (4.8–7.3)	5.3 (4.2–6.5)	3.9 (2.9–4.9)	8.9 (7.2–10)	7.6 (6.2–9.1)	5.3 (4.2–6.9)	14 (12–16)	13 (11–15)	11 (9.4–13)	20 (17–24)	19 (16–22)	16 (14–19)
**Three-day** **Treatment** **(300 µg/kg)**	12 (10–13)	11 (9.3–12)	9.3 (8.1–11)	17 (14–20)	16 (13–18)	13 (11–16)	20 (16–24)	19 (16–22)	17 (14–20)	28 (24–33)	26 (23–31)	24 (21–28)
**Single dose**, **monthly** **(400 µg/kg)**	22 (18–26)	19 (16–23)	15 (12–19)	32 (28–38)	28 (24–34)	22 (17–26)	43 (36–51)	40 (34–48)	35 (30–42)	65 (55–77)	61 (51–72)	53 (45–62)
**Three-day**, **monthly** **(300 µg/kg)**	35 (31–40)	32 (29–37)	28 (25–32)	50 (43–59)	47 (40–54)	40 (34–46)	59 (49–71)	56 (47–67)	51 (43–60)	84 (70- >=90)	80 (67- >=90)	73 (61–86)

Simulations (*n* = 500) were performed up to 90 days. Time > 25%, the number of days where drug concentrations were above those associated with > 25% mosquito mortality. Time > 50%, the number of days where drug concentrations were above those associated with > 50% mosquito mortality. Time > 90%, the number of days where drug concentrations were above those associated with > 90% mosquito mortality. Values are presented as median (25th -75th ). In the monthly administration, 3 rounds of ivermectin were given starting on day 0, day 30 and day 60.

## Discussion

Ivermectin has potential to limit the spread of malaria due to its mosquito-lethal properties^13,15,29^. To fully utilize this potential, it is important to understand the pharmacokinetic-pharmacodynamic properties of ivermectin and its metabolites. In the current work we have successfully used rich pharmacokinetic data in healthy Thai volunteers to establish a well-defined pharmacokinetic model for ivermectin and its three main metabolites, which was linked to mosquito mortality resulting in a dynamic model that can predict the magnitude and duration of mosquito mortality after treatment with ivermectin. This could help in the design of future MDA campaigns which include ivermectin. Simulations from the model confirm a prolonged effect of ivermectin, showing a sustained mosquito mortality (> 90%) for 9.3 days for *An. dirus* and for 17 days for *An. minimus*, after a three-day treatment with ivermectin (300 µg/kg) alone (Table 2; Fig. 5). This suggests that adding ivermectin to antimalarial mass drug administration might help limit the transmission of malaria. Since dihydroartemisinin-piperaquine is well-tolerated, provides prophylactic protection, and has been shown in MDAs to reduce malaria prevalence and incidence in Southeast Asia^30^, we chose to model the interaction of this drug combination on ivermectin pharmacokinetics and mosquito-lethal effect.

The pharmacokinetic model of ivermectin was first developed without any metabolite data. The ivermectin model consisted of a two-compartment model with transit absorption, which was observed previously^31–36^. The initial pharmacokinetic models for the metabolites were estimated using an ivermectin model fixed on an individual level, to increase the stability of the model and help derive the structural models of the metabolites. To describe the pharmacokinetics of metabolite M6 accurately, a direct metabolism pathway from ivermectin was needed (Fig. 5), which improved the description of the formation data of this metabolite. After the structural model for both ivermectin and metabolites had been established, the data for ivermectin and its metabolites were fitted simultaneously. The covariate evaluation showed that concomitant treatment with dihydroartemisinin-piperaquine reduced the elimination clearance of ivermectin, resulting in an increased exposure to ivermectin. This is in line with what was seen in the model-independent analysis of the same data (i.e. non-compartmental analysis)^23^. The inclusion of metabolite data in this analysis provided more information on the elimination clearance of ivermectin, through the formation of metabolites, which might have helped to identify this covariate relationship. A reduction in elimination clearance was also observed in in-vitro drug-drug interaction microsome experiments^37^, most likely due to a competitive interaction with CYP3A4, which is the major enzyme responsible for the metabolism of both ivermectin^25,38^ and piperaquine^39^. This drug-drug interaction has an impact on the mosquito mortality, illustrated in these results by showing a sustained mosquito killing greater than what was seen when ivermectin was administered alone. The covariate analysis also identified a slower absorption of ivermectin when co-administered with dihydroartemisinin-piperaquine as a result of a relatively large increase in mean transit time (32%), this will result in a lower maximum ivermectin concentration. The reason for this change is unknown and need further investigation. However, with high potency to kill mosquitoes this will not have a clinically relevant effect on mosquito mortality. This was not seen in the non-compartmental analysis, which showed no change in T_max_ when co-administrating these drugs. However, nonlinear mixed-effects modelling is better suited to identify true trends and potential differences in the absorption phase since all observed data are fitted with a biologically plausible model. A non-compartmental analysis is only reporting the observed time points associated with highest concentration measurements and the derived parameter T_max_ is therefore highly dependent on the sampling design.

The evaluation of biological sex as covariate showed that the peripheral volume of distribution was different between males and females with a 75% higher volume in females. This is similar to what have been identified before and could be explained by a higher average fat-to-muscle ratio in women^35,40^. This change will result in different half-lives in males and females (2.8 days compared to 4.6 days in women), resulting in prolonged killing of mosquitoes when fed on ivermectin-treated females as can be seen in figure S9.

All metabolites followed formation rate-limited kinetics, resulting in a carry-forward effect of any covariates identified on ivermectin to its metabolite. This also complicates the interpretation of potentially significant covariates identified on metabolites. Thus, the covariate analysis focused on investigated covariates on the pharmacokinetic parameters of ivermectin, and not its metabolites, for simplicity.

The analysis of the metabolites showed that these are present in lower concentrations compared to ivermectin, with metabolite M1 presenting the highest concentration of the three metabolites. Ivermectin and metabolites showed similar pharmacokinetic profiles and terminal elimination half-lives, suggesting a formation rate-limited elimination of metabolites (Figure S8). This indicates that prolonged mosquito killing due to metabolites with longer half-life is not likely. Instead, a combination of all four compounds contribute to the mosquito-lethal effects.

In the final model, the difference between plasma samples and whole blood samples was modeled with a proportional scaling factor on the individual prediction. This showed a higher ivermectin concentration in plasma compared to whole blood (86% higher). This is most likely due to the high plasma protein binding of ivermectin (99.86%)^41^. The M3 and M6 metabolites also showed higher concentration in plasma (76% and 157%, respectively), while M1 showed a 31% lower concentration in plasma.

The pharmacodynamic model was based on cumulative mortality for 10 days after feeding on ivermectin-containing volunteer blood, and showed an effective killing of both *An. dirus* and *An*. *minimus*. A direct effect model with a sigmoidal E_max_ function and the baseline mortality fixed to observed values was applied to describe the relationship between ivermectin exposure and mosquito mortality. The driver of the effect was assumed to be the sum of whole blood concentrations of ivermectin and the three metabolites (Table 1), as these have been demonstrated to have equivalent mosquito-lethal effect in *An. dirus* and *An. minimus*^24^.

In some clinical trial settings, quantification of metabolites might not be possible, so the final model was therefore also re-estimated with only whole blood ivermectin concentrations driving the effect (Table S1). Similarly, whole blood concentrations might not be available so the model was also re-estimated with ivermectin plasma concentrations with and without metabolites driving the effect (Table S1). The developed models did not include any between-subject variability, since each individual blood sample (i.e. ivermectin concentration) was associated with its observed mosquito mortality from an independent ex-vivo experiment. Thus, any variability in the pharmacodynamic parameters would most likely be due to differences between mosquito cohorts and not due to differences between the human individuals. Therefore, the evaluation of the pharmacodynamic variability was instead focused on the residual error model, which was described by a gradual decrease in the relative residual error with increasing ivermectin concentration. This is commonly seen with count data as there is a higher inherent error in a low count observation (baseline mosquito mortality) compared to a higher count observation where most mosquitoes are killed (maximum mortality).

No meaningful differences in ivermectin potency were observed for either species between direct and membrane feeding methods across the four different timepoints. A previous study with *An. gambiae* s.s. found similar results at a single timepoint^42^. This indicates, in ivermectin trials, that mosquito membrane feeding can be used in replacement of mosquito direct feeding. Mosquito membrane feeding is more acceptable from an ethical standpoint and is more accommodating for the trial volunteers.

Differences in ivermectin susceptibility between the *An. dirus* Thai and Cambodian strains were also investigated, but EC_50_ values had overlapping 95% confidence intervals, therefore none were kept in the final model. To our knowledge this is the first trial to directly compare the ivermectin mosquito-lethal effect against two strains of the same mosquito species. These results suggest that ivermectin should be effective throughout the range of a given species unless some populations have inherent metabolic cross-resistance or have developed ivermectin resistance due to repeated prior exposure. To date, no clinical trials have evaluated insecticide-resistant *Anopheles* strains for their susceptibility to ivermectin.

Different dosing scenarios and dose strengths were simulated using the final pharmacokinetic-pharmacodynamic model to evaluate the potential of ivermectin to be used for malaria parasite transmission control. Simulations showed that increasing the number of treatment days resulted in a proportional increase in the overall duration of time that ivermectin achieved mosquito-lethal concentrations in the subjects. This is in contradiction to what was recently reported by Kamau et al., which showed an increased killing of *An. gambiae* s.s. after a single dose at 400 µg/kg compared to three days at 300 µg/kg^43^. In the present study, giving ivermectin over three days will keep the concentration higher for a longer time which should keep killing mosquitoes for longer. A simulated three-day treatment (300 µg/kg) of standard doses of oral ivermectin resulted in 9.3 and 11 days above the concentrations needed to kill 90% and 50% of *An. dirus* mosquitoes. *An. minimus* was substantially more sensitive and the simulated three-day treatment resulted in 17 and 19 days above the concentration needed to kill 90% and 50% of mosquitoes. This study also showed that even higher concentrations of ivermectin are achieved when combining it with dihydroartemisinin-piperaquine, further supporting this combination in MDA for malaria control. The model could also be used to evaluate mosquito mortality after administration of ivermectin with different absorption properties, such as novel long-lasting formulations. The relationship between ivermectin exposure and mosquito killing effects can also be used to parameterize larger population-based malaria transmission models, to fully explore the impact of ivermectin in malaria control and elimination.

The study has several limitations. The study was conducted in a relatively small number of healthy volunteers and the results should be confirmed in a larger patient population. However, it is not expected that the pharmacokinetic properties would differ to a great extent between this Thai population and an endemic population elsewhere in Southeast Asia. The mosquitoes used here are laboratory strains, and might show differences in ivermectin susceptibility compared to wild *Anopheles* mosquitoes with differing genetic backgrounds and insecticide exposure history. Another limitation of this study is the number of *An. minimus* mosquito mortality samples from Study 2, due to the unexpectedly high pre-treatment mortality prompting removal of some individuals from the analysis. However, enough data was retained to allow for a model to be developed. There was also a significant difference in baseline mortality between the two studies, while the colonies were reared in the same insectary, the adults post blood-feeding were maintained in two different laboratories. However, the mosquito mortality experiment inherently includes inter-experiment variability and this was included in the residual variability of the model and did not impact the conclusions.

In summary, a joint pharmacokinetic-pharmacodynamic model was successfully developed describing both the pharmacokinetic properties of ivermectin and its metabolites, as well as linking their exposure to the mortality of two critical malaria vector species. This is the first study describing and quantifying the dynamic relationship between ivermectin dosing, drug exposure and mosquito-killing effects, and will be a useful tool to assess the potential role of ivermectin in malaria transmission control.

## Methods

### Clinical studies

In the present work, data from two different clinical trials were used, both trials were conducted at the Hospital for Tropical Diseases, Faculty of Tropical Medicine, Mahidol University, Bangkok Thailand. The first trial (study 1) was a sequential open label drug-drug interaction trial investigating the interaction between ivermectin and dihydroartemisinin-piperaquine and primaquine, conducted in 16 healthy Thai volunteers. Main inclusion criteria included healthy male and female non-smoking volunteers, aged between 18 and 60 years with body weights between 36 and 75 kg, with normal electrocardiogram and willingness and ability to comply with the protocol were deemed eligible to participate in the trial. Main exclusion criteria included pregnant females, positive for Hepatitis B, Hepatitis C, or HIV at screening, personal history of cardiac, renal or hepatic disease, use of prescription or non-prescription drugs, vitamins, herbs, or supplements especially CYP3A4 inhibitors and inducers, known allergy to any study medication, evidence of active substance abuse, or history of travel to West or Central Africa. A full list of the Inclusion and Exclusion Criteria along with study description can be found here at clinicaltrials.gov (NCT02568098)^23^. The second trial (Study 2) was a pharmacokinetic trial investigating ivermectin and its metabolites when ivermectin was given to 10 healthy Thai volunteers. Main inclusion criteria were the same as for study 1 with the addition that all volunteers needed to be able to tolerate direct mosquito feeding and have a baseline blood hemoglobin above 13 g/dl for males and above 12 g/dl for females. Main exclusion criteria were the same as for Study 1 with the addition of history or suspected of hypersensitivity/anaphylaxis to mosquito bites. A full list of the Inclusion and Exclusion Criteria along with study description can be found at clinicaltrials.gov (NCT03690453). Both studies received human ethical approvals from the ethics committee at the Faculty of Tropical Medicine (Study 1: reference number TMEC 15–004, approval number MUTM 2015-016-02; Study 2: MAL18006, approval number MUTM-2018-069-01), Mahidol University, Bangkok, Thailand; the ethics committee at the University of Oxford (Study 1: OxTREC 4–15; Study 2: OxTREC 33 − 18); and from the institutional review board at the Walter Reed Army Institute of Research (Study 1: WRAIR#2228; Study 2: WRAIR#2609). All participants provided written informed consent before the trials. Study 2 received animal ethical approval from the ethics board at the Faculty of Tropical Medicine (Study 2: reference number 007-2018), Mahidol University, Bangkok, Thailand. Study 1 was performed prior to the requirement of mosquito-based studies to receive animal ethical approval in Thailand. All methods were conducted in accordance with relevant guidelines and regulations.

### Study design

Study 1 was a sequential trial consisting of four arms administering single oral doses of; (1) ivermectin (400 µg/kg), (2) ivermectin (400 µg/kg) given with primaquine, (3) ivermectin (400 µg/kg) given with dihydroartemisinin-piperaquine, and (4) ivermectin (400 µg/kg) given with primaquine and dihydroartemisinin-piperaquine given to healthy Thai volunteers. Study medication was administered with a glass of water 30 min after a similar light standard (low-fat) meal. Study medication was administered under clinical supervision and subjects observed in the hospital for 24 h post-treatment. Subjects were followed up to 36 days after the last dose. The primary outcome was to assess the safety and tolerability of the co-administered drugs, with specific focus on change in QTc prolongation. Primary safety outcomes for Study 1 were published previously^23^. Secondary outcomes were to characterize potential pharmacokinetic interactions and their effect on mosquito-lethal outcomes, and these results are presented in this manuscript. Venous blood samples were collected at 0 (pre-dose), 0.5, 1, 1.5, 2, 3, 4, 6, 8, 12, 24 h after dose, and also on days 2, 3, 6, 10 after dose. Mosquito mortality was investigated for *An. dirus* and *An. minimus* with a membrane feeding assay. Venous whole blood samples for membrane feeding were taken at 0 (pre-dose), 4, 24 h after dose and on days 2, 3, 6, 10 after dose.

Study 2 was designed to study the pharmacokinetics of the metabolites of ivermectin. It was an open-labelled single-arm study in which a single oral dose of ivermectin (400 µg/kg) was given to healthy Thai volunteers. Study medication was administered with a glass of water 30 min after a similar light standard (low-fat) meal. Study medication was administered under clinical supervision and subjects observed in the hospital for 24 h post-treatment. Subjects were followed up to 41 days after the last dose. The primary outcome was to assess the pharmacokinetics and mosquito-lethal effect of ivermectin and metabolites. Secondary outcomes were to characterize the duration of ivermectin mosquito-lethal efficacy in *An. dirus* and *An. minimus*, determine if there were mortality differences between the two *An. dirus* strains, and if there were differences between the direct and membrane feeding exposure. Venous blood samples were collected for drug quantification at 0 (pre-dose), 1, 2, 4, 6, 12, 24 h after dose, and also on days 2, 3, 6, 9, 12, 15, 18, 21, 24, 27, 34, 41 after dose. Mosquito mortality for *An. dirus* and *An. minimus* was investigated with both membrane and direct feeding assays. Whole blood samples for membrane feeding were taken at 0 (pre-dose), 3, 6, 9, 12, 15 days after drug administration for *An. dirus* (Thailand strain), at days 0 (pre-dose), 3, 6, 9, 12, 15 after drug administration for *An. dirus* (Cambodia strain), and at days 0 (pre-dose), 9, 12, 15, 18, 21, 24, 27, 34, and 41 after drug administration for *An. minimus*. Direct feeding of *An. dirus* (Thailand strain) were performed at days 0 (pre-dose), 3, 6, 9, 12 after drug administration and at days 0 (pre-dose), 12, 15, 18, 21 for *An. minimus*.

For the mosquito mortality experiments, mosquitoes were reared as described previously at the Armed Force Research Institute of Medical Sciences, Department of Entomology, Bangkok, Thailand^44^. After blood-feeding, the mosquitoes were kept in incubators at 25 ± 1 °C and 80 ± 10% relative humidity, and a 12:12 h light: dark photoperiod. Post blood-feeding, mosquitoes in study 1 were maintained at AFRIMS while mosquitoes in study 2 were maintained at Mahidol University. Mosquitoes were maintained in 0.5 L cardboard cartons and provided 10% sugar *ad libitum*. Mosquitoes were between 5 and 7 days post emergence at the time of blood feeding, and were sugar-starved with access to water from 12 to 18 h before their blood meal. At each mosquito membrane feed, 1 ml of venous whole blood was provided to groups of mosquitoes via glass membrane feeders sealed with baudruche membrane and warmed to 37 °C by a Lauda Alpha A12 water bath circulator (Lauda Scientific, Lauda-Königshofen, Germany). In study 1, up to 100 mosquitoes were used per membrane feed and 40 blood-fed mosquitoes were retained for mortality monitoring. In study 2, up to 40 mosquitoes were used per membrane feed and 30 blood-fed mosquitoes were retained for mortality monitoring. In study 2, mosquito direct feeding was performed by resting the volunteer arm on top of the 0.5 L cardboard container and covering the arm and container with a towel. Up to 40 mosquitoes were used per direct feed and 30 blood-fed mosquitoes were retained for mortality monitoring. With both methods mosquitoes were allowed up to 30 min to blood feed and afterwards blood-fed females were gently transferred to a clean cardboard container. Post blood meal, mosquito mortality was observed daily, wherein dead mosquitoes were removed and recorded, and any remaining mosquitoes at day 10 were frozen and counted as alive. Mosquito mortality was defined as the total proportion of mosquitoes that were dead at day 10 post feeding.

Kaplan-Meier analysis was performed in GraphPad Prism v10.4.2 based on the observed data which was used in the modelling process (survival up to day 10 after feeding). In the analysis the *An. dirus* and *An. minimus* survival after membrane feeding were assessed to compare Study 1 and Study 2.

### Drug quantification

In both trials, concentrations of ivermectin and its metabolite were quantified using a recently published method^24,28^. Drug was quantified in three separate matrices; venous whole blood, capillary whole blood, and venous plasma. The drug quantification was carried out using high-performance liquid chromatography coupled with a tandem mass-spectrometer. The lower limit of quantification (LLOQ) was 0.2 ng/mL for ivermectin, M1, M3, and M6. Triplicates of quality control samples at low, median, high concentration were analyzed within each batch of clinical samples to ensure precision and accuracy during drug quantification. Quality control samples showed < 15% deviation from true values, which is within the regulatory requirements for bioanalytical methods.

### Pharmacometric analysis - general

Data from the two trials were pooled together and all concentration-time data were transformed into their natural logarithm and used for modelling in Pumas v2.5.1 and 2.6.1^27^. The models were evaluated in terms of objective function as well as general goodness-of-fit. Eta- and epsilon-shrinkage were obtained directly from Pumas AI^45^. Parameter precision was obtained using the sampling important resampling procedure^46^. Due to the long follow-up period in study 2, several ivermectin and metabolite concentrations in the terminal phase were measured to be below the LLOQ. Thus, the first sample in a series of samples below the LLOQ was set to half the quantification limit and the subsequent samples were omitted as missing data (M6 method)^47^.

### Pharmacokinetic modelling of ivermectin

Initially, ivermectin was modelled alone. One-, two-, and three-compartment distribution models were evaluated. To describe the absorption of ivermectin, a first-order absorption model as well as a transit compartment model (1–5 absorption compartments) were evaluated^48^. Relative bioavailability was fixed to 100% with an estimated between-subject variability. Body weight was added using an allometric function centered on 60 kg with fixed exponents (0.75 for clearance parameters and 1 for volume parameters) from the start of the modelling^49,50^. The residual error was modeled with an additive error on the natural logarithm of the predicted concentrations, equivalent to an exponential error on non-logged concentrations.

### Pharmacokinetic modelling of metabolites

In the investigation of the metabolites, the ivermectin model was initially fixed using the final individual pharmacokinetic parameter estimates. The three metabolites (M1, M3, and M6) were evaluated at the same time and it was assumed that ivermectin formed M1 and M3, which in turn was metabolized to M6. A direct metabolism from ivermectin to M6 was also added to be able to describe the observed early concentrations of M6 (estimated only on a population level). What was not metabolized to M6 was metabolized to M1 and M3 in equal proportions, as this parameter was structurally unidentifiable from the observed data. One-, two-, and three-compartment disposition models were investigated for each metabolite. Body weight was added from the start with an allometric function centered on 60 kg with an exponent of 0.75 for clearance parameters and 1 for volume parameters. The residual error was modeled with an additive error on the natural logarithm of the predicted concentrations, equivalent to an exponential error on non-logged concentrations.

### Simultaneous pharmacokinetic modelling of ivermectin and metabolites

The final structural ivermectin and metabolite models were implemented, and data fitted simultaneously. The simultaneous joint model was used to investigate the impact of drug-drug interactions and sex using a stepwise forward covariate search with a significance level of 0.01 in forward step and 0.001 in the backward step. In addition to the statistical criterion, small covariate effects (< 20%) were removed as the clinical significance was deemed low. Concomitant administration of piperaquine and/or primaquine were investigated on the pharmacokinetic parameters of ivermectin. After the identification of drug-drug interactions, sex was investigated as a covariate on ivermectin elimination clearance, central and peripheral volume of distribution, mean transit time, and relative bioavailability.

Simulation-based diagnostics (visual-predictive checks) were performed using 1,000 simulations^51^. Visual-predictive checks were performed in NONMEM, using perl-speaks NONMEM, with the final parameter estimates and model structure fixed to what was obtained in Pumas AI ^52–54^.

### Pharmacokinetic-pharmacodynamic modelling

The final joint pharmacokinetic model of ivermectin and metabolites were used to characterize the pharmacodynamic outcome (i.e., mosquito mortality). The relationship between drug concentration and mosquito mortality was quantified using a sigmoidal E_max_ model as seen in Eq. 1.

1 $$\:E={E}_{0}+\frac{\left({E}_{max}-{E}_{0}\right)\cdot \:{C}^{\gamma\:}}{{EC}_{50}^{\gamma\:}+{C}^{\gamma\:}}$$

The maximum effect (E_max_) was fixed to achieve a total maximum mosquito mortality of 100% and the concentration (EC_50_) that causes 50% mortality (E_max_ +E_0_)/2) and the shape factor (γ) was estimated. The mosquito mortality at baseline (E_0_) was fixed to the individual observed median baseline values, as estimating this parameter resulted in a systematic bias of the baseline mortality. The driver of the mosquito-lethal effect was the sum of the molar venous whole blood concentrations of ivermectin and the three metabolites (C). No between-subject variability was included in the model. Covariates (feeding assay and strain of *An. dirus* mosquito) were investigated in the pharmacodynamic model through a stepwise procedure in the same manner as in pharmacokinetic model. The residual error was modelled with a concentration varying residual error with a smooth transition according to Eq. 2:

2 $$\:\sigma\:={\sigma\:}_{low\_conc}+\frac{{\sigma\:}_{high\_conc}-{\sigma\:}_{low\_conc}}{1+{e}^{-\kappa\:\cdot\:(C-{C}_{cutoff})}}$$

σ is the standard deviation for the final residual error. σ_low_conc_ is the residual error at low concentrations, σ_high_conc_ is residual error at high concentrations, fixed to 0.000001. κ is the slope factor for the smooth transition between the two residual errors, fixed to 1 for stability. C_cutoff_ is the cut-off concentration at which the residual error will change.

Simulation-based diagnostics of the final pharmacokinetic-pharmacodynamic model was performed with a restriction to not allow simulated mosquito mortality over 100% (these were set to 100%) or below 0% (these were set to 0%). Simulation-based diagnostics were performed by simulating the final pharmacokinetic-pharmacodynamic model 1000 time (both the pharmacokinetics and pharmacodynamics were simulated) in NONMEM followed by plotting mortality vs. concentration in GraphPad Prism v10.4.2. For translational purposes, the final pharmacokinetic-pharmacodynamic models for *An. dirus* and *An. minimus* were also re-estimated assuming the following drivers of the pharmacodynamic effect; (1) venous whole blood ivermectin concentrations, (2) venous plasma ivermectin concentrations, and (3) sum of molar venous plasma concentrations of ivermectin and its three metabolites.

### Simulation of different field use cases

The final joint pharmacokinetic-pharmacodynamic model for membrane feeding was used to simulate expected impact of different ivermectin dosing scenarios on transmission control in malaria. Male volunteers were simulated as they have the shortest terminal half-life and therefore representing the worst-case scenario. Different dosing scenarios of ivermectin were simulated; (1) single oral 400 µg/kg dose of ivermectin, (2) single oral 300 µg/kg dose of ivermectin, (3) once daily oral 300 µg/kg dose of ivermectin for three days, (4) single oral 400 µg/kg dose of ivermectin administered once a month for three months (5) once daily oral 300 µg/kg dose of ivermectin for three days administered for three months. All dosing scenarios were assessed both as ivermectin alone and when administered together with dihydroartemisinin-piperaquine, assuming a male volunteer at 60 kg body weight. A total of 1000 simulations (500 for ivermectin alone and 500 then given with dihydroartemisinin-piperaquine) were done per dosing scenario and the mean mosquito mortality and the associated 50% prediction interval was visualized over time. In addition, a single dose of ivermectin (400 µg/kg) were simulated for male and female volunteers (both with a weight of 60 kg) to illustrate any differences in mosquito mortality. Simulations were performed without residual errors. These simulations and plotting were performed in Pumas AI.

## Supplementary Information

Below is the link to the electronic supplementary material.

Supplementary Material 1

## Data Availability

Request for access to data, protocol, and statistical analysis plan can be submitted to the Data Access Committee at Mahidol Oxford Tropical Medicine Research Unit (datasharing@tropmedres.ac). Applications are commonly reviewed within 2 weeks.

## Declarations

### Disclaimer

Material has been reviewed by the Walter Reed Army Institute of Research. There is no objection to its presentation and/or publication. The opinions or assertions contained herein are the private views of the author, and are not to be construed as official, or as reflecting true views of the Department of the Army or the Department of Defense. The investigators have adhered to the policies for protection of human subjects as prescribed in AR 70–25.
