# Supplementary material for: A dynamic pharmacometric framework defining the relationship between ivermectin exposure and mosquito lethality
Source: Sci Rep. 2026 Jul 18;16:32834. doi: 10.1038/s41598-026-58918-9 (PMC13677433; doi:10.1038/s41598-026-58918-9)
Supplement: Supplementary file 1 [file 41598_2026_58918_MOESM1_ESM.docx]

Supplementary material

A dynamic pharmacometric framework defining the relationship between ivermectin exposure and mosquito lethality

*Richard M. Hoglund, Kevin C. Kobylinski, Podjanee Jittamala, Borimas Hanboonkunupakarn, Narenrit Wamaket, Patchara Sriwichai, Siriporn Phasomkusolsil, Nicholas J. White, Nicholas P.J. Day, Joel Tarning*

Table S1. Pharmacokinetic and pharmacodynamic parameters for different drivers of mosquito-killing effect.

| ***An. Dirus*** | | | |
| --- | --- | --- | --- |
|  | **Whole blood**  **Ivermectin** | **Venous plasma**  **Ivermectin** | **Venous plasma**  **Ivermectin+metabolites** |
| E_max_ (%) | 100 *fix* | 100 *fix* | 100 *fix* |
| EC_50_ (nM) | 3.58 | 6.68 | 12.2 |
| γ | 5.72 | 5.86 | 6.25 |
| σ_low_conc_ | 0.184 | 0.185 | 0.182 |
| σ_high_conc_ | 0.000001 *fix* | 0.000001 *fix* | 0.000001 *fix* |
| Κ | 0.5 *fix* | 0.5 *fix* | 0.5 *fix* |
| C_cutoff_ (nM) | 23.8 | 47.2 | 89.7 |

| ***An. Minimus*** | | | | |
| --- | --- | --- | --- | --- |
|  | **Whole blood**  **Ivermectin** | **Venous plasma**  **Ivermectin** | | **Venous plasma**  **Ivermectin+metabolites** |
| E_max_ (%) | 100 *fix* | | 100 *fix* | 100 *fix* |
| EC_50_ (nM) | 0.530 | | 0.985 | 1.98 |
| γ | 4.43 | | 4.72 | 5.55 |
| σ_low_conc_ | 0.118 | | 0.0992 | 0.124 |
| σ_high_conc_ | 0.000001 *fix* | | 0.000001 *fix* | 0.000001 *fix* |
| Κ | 0.5 *fix* | | 0.5 *fix* | 0.5 *fix* |
| C_cutoff_ (nM) | 4.07 | | 11.7 | 16.6 |

Ivermectin+metabolites is when the sum of ivermectin and all metabolites concentrations drives the effect. Ivermectin is when the concentrations of only ivermectin drives the effect. Venous plasma is when the venous plasma concentrations drives the effect. Whole blood is when the whole blood concentrations drive the effect. E_max_ is the maximum effect, EC_50_, is the concentration resulting in 50% of maximum effect, Baseline is the baseline mortality, γ is the shape factor in the sigmoidal E_max_-model. σ_low_conc_ is an additive residual error used at concentrations lower than the cutoff, σ_high_conc_ is the additive residual error used at concentration above the cutoff, Κ is the slope factor in the transition between low and high residual error, C_cutoff_ is the cutoff concentration (sum of molar concentrations of parent and metabolites) for the transition between low and high residual error.

a. Parameter estimates from estimation in Pumas AI. IIVs are presented as variance and σ is presented as standard deviation.

Figure S1. Goodness-of-fit plot for ivermectin. The plot in the top left (A) show population prediction versus observed concentration. The plot in the top right (B) show individual prediction versus observed concentration. The plot in the bottom right (C) show population prediction versus conditional weighted residuals. The plot in the bottom left (D) show time after dose versus conditional weighted residuals. The dashed black line in the LOESS (locally estimated scatterplot smoothing) regression line. The solid black line is line of identity.

Figure S2. Goodness-of-fit plot for 3”-*O*-demethyl ivermectin (M1). The plot in the top left (A) show population prediction versus observed concentration. The plot in the top right (B) show individual prediction versus observed concentration. The plot in the bottom right (C) show population prediction versus conditional weighted residuals. The plot in the bottom left (D) show time after dose versus conditional weighted residuals. The dashed black line in the LOESS (locally estimated scatterplot smoothing) regression line. The solid black line is line of identity.

Figure S3. Goodness-of-fit plot for 4-hydroxyl ivermectin (M3). The plot in the top left (A) show population prediction versus observed concentration. The plot in the top right (B) show individual prediction versus observed concentration. The plot in the bottom right (C) show population prediction versus conditional weighted residuals. The plot in the bottom left (D) show time after dose versus conditional weighted residuals. The dashed black line in the LOESS (locally estimated scatterplot smoothing) regression line. The solid black line is line of identity.

Figure S4. Goodness-of-fit plot for 3”-*O*-demethyl, 4-hydroxyl ivermectin (M6). The plot in the top left (A) show population prediction versus observed concentration. The plot in the top right (B) show individual prediction versus observed concentration. The plot in the bottom right (C) show population prediction versus conditional weighted residuals. The plot in the bottom left (D) show time after dose versus conditional weighted residuals. The dashed black line in the LOESS (locally estimated scatterplot smoothing) regression line. The solid black line is line of identity.

Figure S5. Kaplan-Meier curves illustrating the difference mosquito survival at baseline after membrane feeding between Study 1 (red) and 2 (green) for both An. Dirus and An. Minimus. The solid lines are the estimated survival and the shaded areas are the 95% confidence interval.

Figure S6. Goodness-of-fit plot for *An. dirus* model. The plot on the left (A) show population prediction versus observed concentration. The plot in the middle (B) show population prediction versus conditional weighted residuals. The plot on the right (C)show time after dose versus conditional weighted residuals. The dashed black line in the LOESS (locally estimated scatterplot smoothing) regression line. The solid black line is line of identity.

Figure S7. Goodness-of-fit plot for *An. minimus* model. . The plot on the left (A) show population prediction versus observed concentration. The plot in the middle (B) show population prediction versus conditional weighted residuals. The plot on the right (C)show time after dose versus conditional weighted residuals. The dashed black line in the LOESS (locally estimated scatterplot smoothing) regression line. The solid black line is line of identity.

Figure S8. Simulated population concentration-time curves of ivermectin (red), metabolites 3”-*O*-demethyl ivermectin (M1) (blue), 4-hydroxyl ivermectin (M3) (green), and 3”-*O*-demethyl, 4-hydroxyl ivermectin (M6) (black), for a 60 kg male volunteer after a single dose of 400 µg/kg ivermectin alone.

Figure S9. The impact of biological sex on pharmacokinetics of ivermectin (left) and the mortality of *An. diurs* (middle) and *An. minimus* (right). Simulated population curves for a 60 kg male (solid black line) and female (dashed red line) volunteer after a single dose of 400 µg/kg ivermectin given alone.

```
Supplementary code.
pkpd_final =
@model begin
    @metadata begin
     desc = "IVM and metabolite model"
     timeu = u"hr"
    end
    @param begin
      "Clearance IVM (L/hr)"
```

      tvcl_IVM ∈ RealDomain(lower = 0)

      "Volume IVM (L)"

      tvvc_IVM  ∈ RealDomain(lower = 0)

      "Intercompartmental Clearance IVM (L/hr)"

      tvq_IVM  ∈ RealDomain(lower = 0)

      "Peripheral Volume IVM (L)"

      tvvp_IVM  ∈ RealDomain(lower = 0)

      "Mean transit time IVM (h)"

      tvmtt ∈ RealDomain(lower = 0)

      "Plasma conversion factor IVM"

      plaf_IVM ∈ RealDomain(lower = -1)

      "Clearance M1 (L/hr)"

      tvcl_M1 ∈ RealDomain(lower = 0)

      "Volume M1 (L)"

      tvvc_M1  ∈ RealDomain(lower = 0)

      "Q M1 (L/hr)"

      tvq_M1 ∈ RealDomain(lower = 0)

      "Volume periferal M1 (L)"

      tvvp_M1  ∈ RealDomain(lower = 0)

      "Plasma conversion factor M1"

      plaf_M1 ∈ RealDomain(lower = -1)

      "Clearance M3 (L/hr)"

      tvcl_M3 ∈ RealDomain(lower = 0)

      "Volume M3 (L)"

      tvvc_M3  ∈ RealDomain(lower = 0)

      "Q M3 (L/hr)"

      tvq_M3 ∈ RealDomain(lower = 0)

      "Volume periferal M3 (L)"

      tvvp_M3  ∈ RealDomain(lower = 0)

      "Plasma conversion factor M3"

      plaf_M3 ∈ RealDomain(lower = -1)

      "Clearance M6 (L/hr)"

      tvcl_M6 ∈ RealDomain(lower = 0)

      "Volume M6 (L)"

       tvvc_M6  ∈ RealDomain(lower = 0)

      "Plasma conversion factor M6"

      plaf_M6 ∈ RealDomain(lower = -1)

      "Fraction direct to M6"

       FPS ∈ RealDomain(lower = 0)

       "Piperaquine effect on CL IVM"

       PPQ_CL_IVM_cov ∈ RealDomain(lower = -1)

       "Piperaquine effect on MTT"

       PPQ_MTT_cov    ∈ RealDomain(lower = -1)

       "Sex effect on VP IVM"

       SEX_VP_IVM_cov ∈ RealDomain(lower = -1)

       ######## PD Dirus ###############

       "EC50 (uM)"

        tv_ec50_dirus ∈ RealDomain(lower = 0)

       "Hill"

        tv_hill_dirus ∈ RealDomain(lower = 0)

        ######## PD Minimus ###############

        "EC50 (uM)"

        tv_ec50_minimus ∈ RealDomain(lower = 0)

        "Hill"

        tv_hill_minimus ∈ RealDomain(lower = 0)

```
       """
      - ΩCL_IVM
      - ΩQ_IVM
      - ΩVp_IVM
      - ΩCL_M1
      - ΩVc_M1
      - ΩCL_M3
      - ΩVc_M3
      - ΩCL_M6
      - ΩF1
      """
```

      Ω    ∈ PDiagDomain(9)

      "Proportional RUV IVM"

      σ_a_IVM  ∈ RealDomain(lower = 0.0000001)

      "Proportional RUV M1"

      σ_a_M1  ∈ RealDomain(lower = 0.0000001)

      "Proportional RUV M3"

      σ_a_M3  ∈ RealDomain(lower = 0.0000001)

      "Proportional RUV M6"

      σ_a_M6  ∈ RealDomain(lower = 0.0000001)

      "Additive RUV Dirus"

      σ_lowconc_dir ∈ RealDomain(lower=0)  # Residual error below cut-off

      cutoff_dir ∈ RealDomain(lower=0.1) # Concentration cut-off

      "Additive RUV Minimus"

      σ_lowconc_min ∈ RealDomain(lower=0)  # Residual error below cut-off

      cutoff_min ∈ RealDomain(lower=0.1) # Concentration cut-off

```
  end
    @random begin
      η    ~ MvNormal(Ω)
        end

    @covariates begin
      "Body Weight"
      BW
      "Sex"
      SEX
      "Concomitant DHA-piperaquine treatment"
      PPQ
      "Plasma samples"
      PLACON
      "ARM"
      ARM
      "Assay"
      FASSAY

    end
     @pre begin
      CL = tvcl_IVM*(1+PPQ_CL_IVM_cov*PPQ) *((BW/60)^0.75)  * exp(η[1])
      Vc = tvvc_IVM *((BW/60)^1)
      Q  = tvq_IVM  *((BW/60)^0.75)
      Vp = tvvp_IVM*(1+SEX_VP_IVM_cov*SEX) *((BW/60)^1)     * exp(η[2])
      MTT = tvmtt*(1+PPQ_MTT_cov*PPQ)                       * exp(η[3])


      CL_M1 = tvcl_M1 *((BW/60)^0.75)                       * exp(η[4])
      Vc_M1 = tvvc_M1 *((BW/60)^1)                          * exp(η[5])
      Q_M1 = tvq_M1   *((BW/60)^0.75)
      Vp_M1 = tvvp_M1 *((BW/60)^1)

      CL_M3 = tvcl_M3 *((BW/60)^0.75)                       * exp(η[6])
      Vc_M3 = tvvc_M3 *((BW/60)^1)                          * exp(η[7])
      Q_M3 = tvq_M3   *((BW/60)^0.75)
      Vp_M3 = tvvp_M3 *((BW/60)^1)

      CL_M6 = tvcl_M6 *((BW/60)^0.75)                       * exp(η[8])
      Vc_M6 = tvvc_M6 *((BW/60)^1)

      PLAEFF_IVM = (1+plaf_IVM*PLACON)

      PLAEFF_M1 = (1+plaf_M1*PLACON)

      PLAEFF_M3 = (1+plaf_M3*PLACON)

      PLAEFF_M6 = (1+plaf_M6*PLACON)

      nn=3
      KTR = (nn+1)/MTT

      K14=KTR
      K45=KTR
      K56=KTR
      K62=KTR
      K27=(1-FPS)*0.5*CL/Vc
      K28=(1-FPS)*0.5*CL/Vc
      K29=FPS*CL/Vc
      K23=Q/Vc
      K32=Q/Vp
      K79 = CL_M1/Vc_M1
      K7T10 = Q_M1/Vc_M1
      K10T7 = Q_M1/Vp_M1
      K89 = CL_M3/Vc_M3
      K8T11 = Q_M3/Vc_M3
      K11T8 = Q_M3/Vp_M3
      K90 = CL_M6/Vc_M6

      DEL=0.00001
      ############## PD Dirus ############
      EC50_dirus = tv_ec50_dirus
      Hill_dirus = tv_hill_dirus
      BL_dirus   = 0.03735

      ############## PD Minimus ############
      EC50_minimus = tv_ec50_minimus
      Hill_minimus = tv_hill_minimus
      BL_minimus   = 0.0818
    end
    @dosecontrol begin
      bioav = ( ; A1=1 * exp(η[9]))

    end
    @dynamics begin
      A1'      = -K14*A1
      A2'      =  K62*A6+ K32*A3 -K23*A2-K27*A2-K28*A2-K29*A2
      A3'      =  K23*A2-K32*A3
      A4'      =  K14*A1-K45*A4
      A5'      =  K45*A4-K56*A5
      A6'      =  K56*A5-K62*A6

      A7'      =  K27*A2+K10T7*A10-K7T10*A7-K79*A7
      A8'      =  K28*A2+K11T8*A11-K8T11*A8-K89*A8
      A9'      =  K29*A2+K79*A7+K89*A8-K90*A9
      A10'      =  K7T10*A7-K10T7*A10
      A11'      =  K8T11*A8-K11T8*A11

    end
    @derived begin
      cp_IVM := @. (A2/(Vc))*(PLAEFF_IVM)
      cp_M1  := @. (A7/(Vc_M1))*(PLAEFF_M1)
      cp_M3  := @. (A8/(Vc_M3))*(PLAEFF_M3)
      cp_M6  := @. (A9/(Vc_M6))*(PLAEFF_M6)
      cp_eff =  cp_IVM+cp_M1+cp_M3+cp_M6

      lcp_IVM := @. log((A2/(Vc))*(PLAEFF_IVM)+0.0000001)
      lcp_M1  := @. log((A7/(Vc_M1))*(PLAEFF_M1)+0.0000001)
      lcp_M3  := @. log((A8/(Vc_M3))*(PLAEFF_M3)+0.0000001)
      lcp_M6  := @. log((A9/(Vc_M6))*(PLAEFF_M6)+0.0000001)

      eff_dirus := @. BL_dirus+(((1-BL_dirus)*(cp_eff+DEL)^Hill_dirus)/(((EC50_dirus+DEL)^Hill_dirus)+((cp_eff+DEL)^Hill_dirus)))
      eff_minimus := @. BL_minimus+(((1-BL_minimus)*(cp_eff+DEL)^Hill_minimus)/(((EC50_minimus+DEL)^Hill_minimus)+((cp_eff+DEL)^Hill_minimus)))

      NDV    ~ @. LogNormal(lcp_IVM, σ_a_IVM)
      NDV_M1 ~ @. LogNormal(lcp_M1, σ_a_M1)
      NDV_M3 ~ @. LogNormal(lcp_M3, σ_a_M3)
      NDV_M6 ~ @. LogNormal(lcp_M6, σ_a_M6)

      k = 1
      σ_highconc = 0.000001

      σ_dir = @. σ_lowconc_dir + (σ_highconc - σ_lowconc_dir) / (1 + exp(-k * (cp_eff - cutoff_dir)))
      σ_min = @. σ_lowconc_min + (σ_highconc - σ_lowconc_min) / (1 + exp(-k * (cp_eff - cutoff_min)))

      DV_MK_Dirus    ~ @. Normal(eff_dirus, σ_dir)
      DV_MK_Minimus    ~ @. Normal(eff_minimus, σ_min)
    end
end


pkpdparams_sim = (tvcl_IVM = 12.265,
                tvvc_IVM  = 211.19,
                tvq_IVM   = 17.655,
                tvvp_IVM  = 616.33,
                tvmtt  = 3.6639,
                plaf_IVM = 0.86379,
                tvcl_M1 = 7.9437,
                tvvc_M1  = 29.063,
                tvq_M1 = 19.21,
                tvvp_M1  = 110.62,
                plaf_M1 = -0.31091,
                tvcl_M3 = 10.25,
                tvvc_M3  = 21.628,
                tvq_M3 = 12.347,
                tvvp_M3  = 94.007,
                plaf_M3 = 0.75674,
                tvcl_M6 = 157.89,
                tvvc_M6  = 93.77,
                plaf_M6 = 1.572,
                FPS = 0.23941,
                PPQ_CL_IVM_cov = -0.40995,
                PPQ_MTT_cov = 0.32282,
                SEX_VP_IVM_cov = 0.74614,
                tv_ec50_dirus = 7.889,
                tv_hill_dirus  = 6.0497,
                tv_ec50_minimus = 1.2929,
                tv_hill_minimus  = 5.5006,
                Ω = Diagonal([0.035104,0.11933,0.055861,0.25786,0.79699,0.078427,0.12605,0.1584,0.07126]),
                σ_a_IVM = 0.51099,
                σ_a_M1 = 0.54613,
                σ_a_M3 = 0.41653,
                σ_a_M6 = 0.44838,
                σ_lowconc_dir = 0.1843,
                cutoff_dir =58.591,
                σ_lowconc_min = 0.14433,
                cutoff_min =7.5613)
```
